# Dehydration and rehydration affect brain regional density and homogeneity among young male adults, determined *via* magnetic resonance imaging: A pilot self-control trial

**DOI:** 10.3389/fnut.2022.906088

**Published:** 2022-09-23

**Authors:** Na Zhang, Jianfen Zhang, Songming Du, Guansheng Ma

**Affiliations:** ^1^Department of Nutrition and Food Hygiene, School of Public Health, Peking University, Beijing, China; ^2^Laboratory of Toxicological Research and Risk Assessment for Food Safety, Peking University, Beijing, China; ^3^Chinese Nutrition Society, Beijing, China

**Keywords:** water, hydration, magnetic resonance imaging, brain structure, connectivity of nodes

## Abstract

The effects of dehydration and rehydration on brain regional density and homogeneity are unknown and have been infrequently studied. In this pilot self-control study, twelve participants aged 18-25 years were recruited and the brain was scanned using magnetic resonance imaging for three tests under different hydration statuses. In three tests, urine osmolality was determined to assess hydration status. Test 1 was conducted after 12 h of overnight fasting. Test 2 was conducted in a dehydration state induced by 36 h of water deprivation. Test 3 was conducted in a rehydration state, which was induced by 1.5 L of purified water supplementation. Compared with test 1, participants under the dehydration state in test 2 had higher cerebrospinal fluid density (*p* < 0.001). Compared with test 2, participants under the rehydration state in test 3 showed an extensive increase in gray matter density in widespread brain regions, mainly involving the left middle temporal gyrus, cuneus, right thalamus, left rolandic opercula, Brodmann area 39, right precentral, left postcentral gyrus, and cingulate gyrus (*p* < 0.001); a higher white matter density in the temporal lobe, sub-lobar, and sub-gyral areas; and a lower cerebrospinal fluid density (*p* < 0.001). The multimodal, multiscale neuroimaging marker of the human brain connection—the regional homogeneity (ReHo) index—was used for evaluating the connectivity of nodes in the brain. Compared with test 1, participants in test 2 had a lower ReHo value in the right amygdala, left occiput median, right lingual, opercula part of right inferior frontal gyrus, and right precuneus (*p* < 0.01). Compared with test 2, participants in test 3 had a higher ReHo value in the right amygdala, right lingual, opercula part of the right inferior frontal gyrus, and right precuneus (*p* < 0.01). Dehydration state increased cerebrospinal fluid density, decreased brain regional homogeneity. Rehydration state increased brain gray matter and white matter density widespreadly, and increased brain regional homogeneity.

## Introduction

Water, as an important nutrient, is essential for the survival and development of life (1). Water plays important roles in various physiological processes, including maintaining the normal osmotic pressure and electrolyte balance of the body fluid, participating in the metabolism of the body, regulating body temperature, etc. (1). Maintaining adequate water intake is vital in ensuring normal physiological functions (1). Insufficient water intake has negative impacts on cognitive performance and physical activity, and it also increases the risk of urinary and cardiovascular diseases (2, 3). Some surveys in China about fluid intake showed that a large proportion of adults, and middle and primary school children did not drink the recommended amount of water, and about a quarter were in a dehydration state, which was judged using the standard of 24 h urine osmolality exceeding 800 mOsm/kg (4–6). According to the results of surveys on water intake in some other countries, the dehydration state is also a common phenomenon due to insufficient water intake in daily life around the world (7–9). However, not enough attention has been paid to the importance of water to health.

The water content of the brain reaches 75% of the brain mass. The water content of the brain’s white and gray matter reaches approximately 70 and 82% in brain white and gray matter mass, respectively (1). The sensation of thirst is the basic instinct to acquire water to maintain a normal hydration state (24 h urine osmolality ≤ 800 mOsm/kg) (10). The sensation of thirst is caused by increased osmotic pressure in the internal environment (1). The hypothalamus is the receptor and regulatory center of osmotic pressure (1). When osmotic pressure rises, the hypothalamus first receives the stimulation but does not produce the sensation of thirst (1). Then, it passes through the afferent nerve to the cerebral cortex, which triggers the sensation of thirst. In the brain, the Na(+) concentration, plasma osmolality, and cerebrospinal fluid (CSF) are continuously monitored to adjust body-fluid homeostasis (11). In addition, the brain also participates in the control of water intake behaviors (1). When the human body feels thirsty, activations of the subfornical organ, orbitofrontal cortex, and pregenual anterior cingulate cortex are involved in generating a pleasant subjective sensation in response to water intake (12, 13). Thus, it is hypothesized that the brain’s structure, brain regional density and homogeneity may be susceptible to changes in the hydration state induced by water intake and water loss.

A few studies have explored the effects of water intake and hydration on brain structure and function and achieved inconsistent results. In a study in 2011, 10 healthy adolescents were recruited, and a dehydration state (urine osmolality > 675 mOsm/kg) was induced by a thermal exercise protocol. The results of brain magnetic resonance imaging (MRI) in the study showed that the blood-oxygen-level-dependent (BOLD) response in the fronto-parietal area was stronger under a dehydrated state (14). It is speculated that the reason for this result was that neuronal activity was higher when dehydrated (14). In a study in 2014, the brains of ten healthy adult participants were scanned using MRI under the dehydration and rehydration states. Additionally, the dehydration state was caused by 14 h of fasting. The rehydration state was reduced after 1.5L of water supplementation. The results showed that the spinal cord cross-sectional area (CSA) decreased under the dehydration state (15). In 2005, a study was conducted among 20 healthy adults, and its results showed that the dehydration state induced after water restriction for 16 h led to a decrease of 0.55% in the brain’s volume. Meanwhile, rehydration after 1.5 L of mineral water supplementation led to an increase of 0.72% in the brain’s volume (16). In a study in 2016, the brains of 20 healthy adults were scanned with MRI under a dehydration state after 9 h of overnight fasting and under a rehydration state after 3L of water was consumed over 12 h (17). The results indicated that no statistical changes were found for brain total water content and brain volume under different hydration states (17). One more study in 2016 found that the dehydration state induced by exercise without replacing fluid losses reduced total brain volume among 10 sportsmen (18). There was one study that investigated the changes in brain structure under the dehydration state using voxel-based morphology, and it showed that there were associations between the decrease in gray matter (GM) and white matter (WM) volume and the dehydrated state in various brain regions (19). In a study conducted among nine physically active adult participants aged 24 years old, it was found that a dehydration state induced by exercise and heat stress with 2.8% body mass loss decreased intracranial volume, reduced subcortical gray matter volume, and expanded the ventricle and cerebrospinal fluid volumes (20). In a long-term hydration experiment, six healthy young adults 25 years old were recruited, and a dehydration state was induced in two days by water restriction to 150 mL water per day (19). Related studies are few, related studies has been summarized in Supplementary Table 1. It is meaningful to conduct further studies to explore the effects of the hydration state on the brain structure and functions using the method of brain magnetic resonance imaging.

The purposes of this study are, first, to analyze the effects of slowly progressive dehydration after 36 h of water deprivation on brain regional density and homogeneity using the method of MRI and, second, to explore the effects of rehydration after an adequate amount of water supplementation on brain regional density and homogeneity among healthy young adults in China. The results of MRI in this study provide more evidence about the importance of hydration. It is also meaningful to bring attention to drinking an adequate amount of water and maintaining an optimal hydration state.

## Materials and methods

### Participants

Twelve healthy male young adults were recruited from one college in Cangzhou, China.

The inclusion criteria were as follows: the age of participants was between 18 and 25 years; the participants were in a healthy state. The exclusion criteria were as follows: the age of participants were <18 years or >25 years; the participants have a history of smoking, habitually consume a large amount of alcohol (>20 g/day), or perform intensive physical exercise (> 6 METs); or they have diseases of the gastrointestinal tract or of the kidney, cognitive disorders, or other chronic and metabolic diseases.

### Sample size calculation

Based on the formula$N={\left[\frac{\left(\alpha +\beta \right)\,\sigma_{d}}{\delta }\right]}^{2}$, to achieve a power (1-beta) of 0.9 with alpha = 0.05, sigma_d = 1.4 and delta = 1.17, 12 subjects were required. Here, sigma_d = 1.4 and delta = 1.17 were based on reference (17). This sample size was also consistent with previous studies in which the sample size was in the range of 6-20.

### Ethics

The study protocol and instruments were reviewed and approved by the Ethical Review Committee of Chinese Nutrition Society on November 10, 2015. The code of identification is CNS-2015-001. The study was conducted in accordance with the guidelines of the Declaration of Helsinki. Prior to the conduction of the study, all participants read and signed informed consent voluntarily.

### Study design and procedure

**FIGURE 1:**
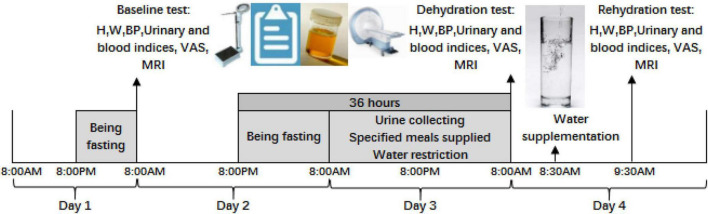
Procedure of the study. H is the abbreviation for height; W is weight; BP is blood pressure; VAS is visual analog scales; MRI is magnetic resonance imaging.

The study procedure of the self-control trial is shown in Figure 1. During the study, volunteers were asked to not perform vigorous-intensity physical activities (e.g., running, race-walking, hiking uphill, etc.), to not smoke or drink alcohol, and to not consume any kinds of caffeine-containing beverages. All participants were monitored by research supervisors and investigators. Three MRI tests were performed, including test 1 under baseline state, test 2 under dehydration state caused by 36 hours of water deprivation, and test 3 under rehydration state after water supplementation. All tests were conducted in Cangzhou Central Hospital.

Day 1: All participants fasted overnight from 8:00 p.m. and were told to sleep no later than 11 p.m. They were required to not urinate until awaking on day 2.

Day 2: First, urine samples were collected at 8:00 a.m. in the morning using a sterile urine sample accumulator and then sent to be tested by lab technicians in the hospital. Cubital venous blood was collected and sent to determine the blood osmolality and blood glucose. Body measurement and blood pressure were also conducted. Visual analog scales on thirst were conducted, and brain magnetic resonance imaging (MRI) was performed as baseline test 1. After test 1 under the baseline state, participants could eat and drink. After 8:00 p.m., participants were required to fast without any food and water for 12 h.

Day 3: Participants could not drink any fluid and lasted for 24 h from 8:00 a.m. on day 3. Three specified solid meals were supplied to participants by a researcher at 7:00 a.m., for breakfast; at 12:00 a.m., for lunch; and at 5:30 p.m., for dinner. No other food was eaten. Fluid intake from foods was assessed using methods of weighing, duplicate portion, and laboratory analysis. Each urine sample was collected by the participants and then sent for evaluation of the 24 h urine volume by researchers. The urine osmolality of each urine sample was also determined. Participants were required to sleep no later than 11 p.m. and to not urinate until awaking on day 4.

Day 4: At 8:00 a.m., test 2 under dehydration state, the same procedure as test 1, was conducted on participants. Brain magnetic resonance imaging was performed under dehydration state caused by 36 h of water deprivation. Participants drank 1,500 mL of purified water in fifteen minutes at 8:30 a.m. and were required to drink 500 mL every 5 min. After resting for an hour, test 3 under rehydration state was performed. Brain magnetic resonance imaging was performed under rehydration state after water supplementation.

The temperature and humidity of the living environment among participants during these days were measured and noted. In the whole process of the study, participants who failed to meet these requirements needed to let investigators know. Finally, all participants finished the study, and no one failed to meet the requirements.

### Definition of hydration state

The standard for the dehydration state was the urine osmolality exceeding 800 mOsm/kg (21). Optimal hydration state was judged in accordance with the standard of urine osmolality less than or equal to 500 mOsm/kg (22). When urine osmolality was more than 500 mOsm/kg and less than or equal to 800 mOsm/kg, it can be judged as the middle hydration state (6). The rehydration state after water supplementation was defined as a urine osmolality less than or equal to 800 mOsm/kg (21).

### Assessment of water intake from foods

Weighing, duplicate portion, and laboratory analysis methods were used to assess water intake from foods. See (23) for the specific steps and calculation methods.

### Anthropometric measurements

Wearing light clothing and no footwear, height (H) was measured twice with 0.1 cm accuracy and weight (W) was measured twice with 0.1 kg accuracy by trained investigators using a height–weight meter (HDM-300; Huaju, Yiwu, Zhejiang, China).

Blood pressure (BP) was measured twice with 2 mmHg accuracy by a nurse with electronic sphygmomanometer (HEM-7051; Omrom, Dalian, Liaoning, China). Two measurements were conducted after 2 min intervals.

BMI (Body Mass Index) = weight (kg)/height squared (m^2^).

### Tests of urine biomarkers

Random spot urine samples were collected in disposable urine storage bags by participants, and then, the samples were stored at +4°C. Starting with the second voiding on day 3 and ending with the first voiding on day 4, all urine samples were collected as total 24 h urine volume on day 3. Urine volume was measured with the accuracy 0.1 kg with electronic desktop scale (YP20001; SPC; Shanghai, China). Additionally, urine osmolality was tested using an osmotic pressure molar concentration meter (SMC 30C; Tianhe, Tianjin, China).

### Assessment of blood biomarkers

Cubital venous blood was also used to test osmolality and glucose of blood. Blood osmolality was tested with an osmotic pressure molar concentration meter (SMC 30C; Tianhe, Tianjin, China). Blood glucose was tested with an automatic biochemical analyzer (Cobas C501; Roche, Basel, Switzerland).

### Assessment of subjective thirst sensation

Visual analog scales (VAS) are a self-rated 10 cm line designed to quantitatively measure the subjective feeling of thirst (24). The labels “not at all” and “extremely” were anchored at the beginning of the line and its end. Participants were required to draw a vertical line corresponding to their degree of thirst. The range of scores for thirst varied between 0 and 10.

### Magnetic resonance imaging scans

Magnetic resonance imaging scans were administered on a 3-teslas SIGNA HDx scanner (Discovery MR 750, General Electric; Milwaukee, WI). Participants laid flat on the scanning stage. The heads of participants were placed centrally, the mandibular was adducted, and intracranial anterior commissure and posterior commissure (AC-PC) line were as parallel as possible to the axial line. If necessary, the localization of head was realigned. Participants were required to stay awake, to close their eyes, to breathe quietly, and to plug their ears with a rubber stopper to reduce noise interference.

Scout image: First, the scout images were acquired by setting a sequence with parameters TE (time of echo) = 1.6 ms, slice number = 5, slice sickness = 7 mm, FOV (field of view) = 30 mm × 30 mm, matrix = 288 × 128, and NEX (number of excitations) = 1.

Structural MRI: Based on the scout images, structural MRI was performed in parallel with the AC-PC line. The sequence of 3D BRAVO was used with parameters TR (repetition time) = 8.2 ms, TE = 3.2 ms, slice number = 132, slice sickness = 1.2 mm, spacing = 0, flip angle = 12°, FOV = 240 mm × 240 mm, matrix = 256 × 256, NEX = 1, and bandwidth = 31.25.

### Temperature and humidity

The temperature and humidity indoor and outdoor were recorded at 10:00 a.m., 2:00 p.m., and 8:00 p.m. with a temperature hygrometer by researchers during the experiment.

### Analysis of structural MRI

Data processing of structural MRI was carried out on the network platform of MATLAB (2012a, MathWorks, Natick, MA, USA). VBM (voxel-based morphometry) of the T1 image was analyzed using the neuroimaging computing software SPM8 (Statistical Parametric Mapping^1^) with toolboxes of VBM8 and DARTEL. The process mainly included the following steps: (a) correction, in which the T1 images were reoriented and calibrated to ensure that the anterior commissure was the origin (0,0,0); (b) segmentation, in which the T1 images after the original point correction were segmented into GM (gray matter), WM (white matter), and CSF (cerebrospinal fluid) voxel fraction images; (c) template generation, in which the group template was generated using the DARTEL method (25) and iterated several times; (d) normalization, in which all images of participants were spatially normalized by registration to the Montreal Neurological Institute brain template (MNI152) and the voxel size after registration was 1.5 mm × 1.5 mm × 1.5 mm; and (e) smoothing, in which the smoothing kernel with 8 mm FWHM (full-width at half maximum) was used to smooth the registered GM, WM, and CSF images. The images for the location of brain regions with statistical differences between the two groups were presented by conventional axial bitmap using Software MRICron^2^ and BrainNet Viewer^3^.

### Analysis of functional MRI in resting state (rs-fMRI)

Data processing of fMRI was also carried out on the platform of MATLAB. SPM8 software toolkit was used for data preprocessing. The processing steps were as follows: (a) the data of the first ten time points were removed to ensure data quality and magnetic balance; (b) slice timing correction, in which due to the protocols of fMRI acquisition, slices in the acquisition plane were not acquired simultaneously or sequentially and, thus, slice timing was corrected for this temporal misalignment; (c) realignment, in which realignment strategies were implemented by aligning each image in the time series to the first reference image, and the subjects were excluded if the head was translated by more than 2 mm or rotated more than 2°; (d) covariates, in which analyses were performed by treating WM, CSF, and other signals that were not related to GM as covariates; (e) normalization, in which spatial normalization of the fMRI images was carried out for the differences in anatomical structure, all images were spatially normalized by registration to the MNI152 template, and the size of the voxel after registration was 3 mm × 3 mm × 3 mm; and (f) analysis of regional homogeneity (ReHo) was performed with DPABI software^4^ (26).

### Statistical analysis

SAS 9.2 (SAS Institute Inc., Cary, NC, USA) was used. The mean and standard deviation (SD) were used to describe the quantitative parameters; count data (hydration state) were presented as n (percentage). The differences in brain gray matter, brain white matter, and cerebrospinal fluid among brain areas were calculated using SPM8software. The method of one-way analysis of variance (ANOVA) with replicate measures was used to compare the quantitative parameters among test 1, test 2, and test 3. Then the obtained differential brain regions are subjected to multiple comparison correction (FDR corrected). Finally, the differential brain regions corrected by multiple comparison were used as a mask for post hoc test. The significance levels were set at 0.05 (*p* < 0.05, voxel cluster > = 10). The classification data such as the distribution of hydration state were compared using the method of Chi-square test. When the conditions were not suitable for Chi-square test, such as the expected frequency was less than 5, Fisher exact test was used for comparison and analysis. Significance levels were set at 0.05.

## Results

### Participants characteristics and the environment

All participants finished the study. The average age of these 12 male young adults was 20.8 years, ranging from 19.2 to 23.7 years. The height, weight, BMI, and systolic and diastolic pressures under test 1 were 176.0 ± 5.5 cm, 68.0 ± 10.9 kg, 21.9 ± 3.0 kg/m2, 114.3 mmHg, and 75.1 mmHg (Supplementary Table 2). However, there was statistical significance in blood glucose when compared between test 1, test 2, and test 3 (4.3 ± 0.3 vs. 4.5 ± 0.4 vs. 4.9 ± 0.2, mmol/L; *F* = 11.67, *p* < 0.001), and blood glucose among participants in test 2 was lower than that in test 3. The average temperature of day 1 to 4 was 16.2°C indoors and 20.4°C outside, and the humidity was 32% indoors and 33% outside.

### Hydration state, thirst, and related urine, and blood biomarkers

Among 12 participants, the average water intake from food was 939 ± 146 ml. The 24 h urine volume of participants was 799 ± 145 ml. The void number was 5 ± 2 on day 3 (Supplementary Table 3). The urine osmolality was 1,004 ± 163 (mOsm/kg). Nine participants (75%) were in the dehydration state for the whole day (Supplementary Figure 1).

Statistically, significance was found in blood and urine osmolality and the thirst when compared among three tests. Compared with test 1, the urine osmolality and thirst scores in test 2 were higher, with statistical significance (F = 32.8, *p* < 0.01; *F* = 19.62, *p* = 0.001). Compared with test 2, participants in test 3 had a lower thirst score, urine osmolality and blood osmolality (*F* = 27.64, *p* < 0.001; *F* = 100.95, *p* < 0.001; *F* = 23.31, *p* = 0.001). There was also statistical significance in the distribution of hydration state in three tests (χ2 = 31.270, *p* < 0.001). Compared with test 1, more proportion of dehydration was found in test 2 (50 *vs* 100%). Compared with test 2, less proportion of dehydration was found in test 3 (100 *vs* 8.3%) (Table 1).

**TABLE 1:** Biomarkers related to the hydration state of participants.

	Test 1	Test 2	Test 3
Blood osmolality (mOsm/kg)	304.6 ± 7.1	305.7 ± 6.4	295.3 ± 7.8^#^
Urine osmolality (mOsm/kg)	803.2 ± 171.7*	1123.3 ± 65.7	387.0 ± 268.3^#^
Thirst	3.3 ± 2.2 *	6.8 ± 2.6	1.9 ± 1.5^#^
**Hydration state**			
Dehydration state	6 (50.0%) ^a^	12 (100.0%)	1 (8.3%)
Optimal hydration state	0 (0.0%)	0 (0.0%)	9 (75.0%)
Middle hydration state	6 (50.0%)	0 (0.0%)	2 (16.7%)

*, Statistically significant differences between test 1 and test 2, *P* < 0.025. ^#^, Statistically significant differences between test 2 and test 3, *P* < 0.025. ^a^, Statistically significant differences was found in the distribution of hydration state in three tests when compared with the method of Fisher exact test.

### Changes of brain gray matter density in different hydration states

**FIGURE 2:**
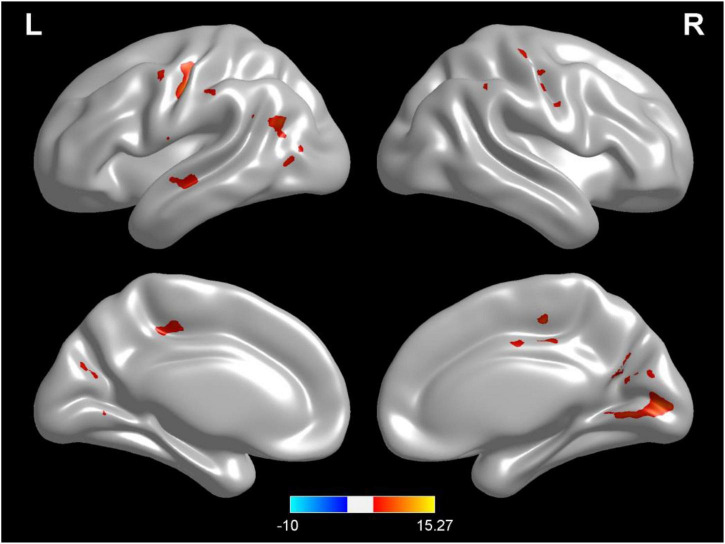
Regional changes on the voxel-based three-dimensional displayed brain gray matter among participants when compared with test 3 in rehydration state and test 2 in dehydration state. Warm colors mean that the gray matter density among participants in the rehydration test was higher than that in the dehydration test; cold colors mean that the gray matter density among participants in the rehydration test was lower than that in the dehydration test; *p* < 0.05 after false-discovery rate correction, voxel threshold of cluster >10.

Compared with test 2, participants in the rehydration state after water supplementation in test 3 showed an extensive increase in gray matter density in widespread brain regions, mainly involving the left middle temporal gyrus, cuneus, right thalamus, left rolandic opercula, Brodmann area 39, right precentral, left postcentral gyrus, and cingulate gyrus (*p* < 0.001) (Table 2 and Figure 2).

**TABLE 2:** Differences in brain gray matter among participants in rehydration state in test 3 and participants in dehydration state in test 2.

Brain areas	Voxel	T	*P*	MNI coordinates		
Temporal_Mid_L (aal)	249	7.25	< 0.001	−48	−16.5	−12
Lentiform Nucleus	55	7.53	< 0.001	16.5	−1.5	−6
Cuneus	1655	11.11	< 0.001	13.5	−69	1.5
Lingual_L (aal)	141	6.12	< 0.001	−13.5	−75	3
Occipital_Mid_L (aal)	117	6.28	< 0.001	−48	−67.5	1.5
Thalamus_R (aal)	413	8.17	< 0.001	9	−12	7.5
Rolandic_Oper_L (aal)	463	15.27	< 0.001	−45	−12	7.5
Precentral Gyrus	56	7.29	< 0.001	−61.5	−4.5	13.5
Transverse Temporal Gyrus	67	6.74	< 0.001	57	−21	12
Brodmann area 39	386	9.88	< 0.001	−49.5	−67.5	22.5
Frontal_Sup_Medial_L (aal)	63	8.30	< 0.001	1.5	45	24
Cuneus_L (aal)	202	6.02	< 0.001	−9	−76.5	27
Postcentral_R (aal)	54	5.55	< 0.001	64.5	1.5	30
Sub-Gyral	192	6.58	< 0.001	−31.5	−78	21
Frontal_Inf_Oper_L (aal)	21	5.35	< 0.001	−42	6	22.5
SupraMarginal_L (aal)	24	6.31	< 0.001	−52.5	−48	27
Precentral_R (aal)	412	7.34	< 0.001	45	−15	51
Postcentral_L (aal)	767	11.19	< 0.001	−43.5	−15	36
SupraMarginal_R (aal)	143	8.88	< 0.001	51	−43.5	40.5
Parietal_Inf_L (aal)	109	5.82	< 0.001	−54	−24	37.5
Cingulate Gyrus	1186	9.10	< 0.001	9	−22.5	49.5
Precuneus	39	7.64	< 0.001	−24	−63	39
Precentral_L (aal)	80	9.25	< 0.001	−46.5	0	49.5
Inferior Parietal Lobule	40	6.54	< 0.001	−42	−54	51

MNI is the abbreviation of Montreal Neurological Institute.

### Changes in brain white matter density in different hydration states

**FIGURE 3:**
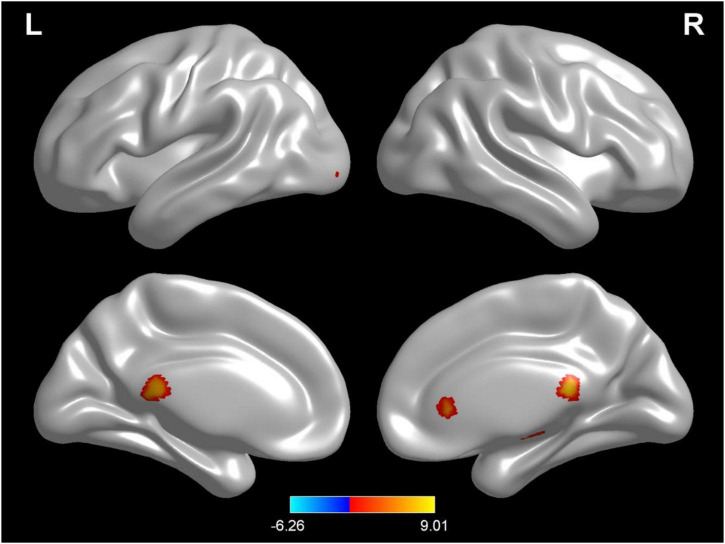
Regional changes on the voxel-based three-dimensional displayed brain white matter among participants when compared with test 3 in rehydration state and test 2 in dehydration state. Warm colors mean that the white matter density among participants in the rehydration test was higher than that in the dehydration test; cold colors mean that the white matter density among participants in the rehydration test was lower than that in the dehydration test; *p* < 0.05 after false-discovery rate correction, voxel threshold of cluster >10.

Compared with test 2, participants in a rehydration state after water supplementation in test 3 had higher white matter density in the temporal lobe, sub-lobar, and sub-gyral (*p* < 0.001) (Table 3 and Figure 3).

**TABLE 3:** Differences in brain white matter among participants in the rehydration state in test 3 and participants in the dehydration state in test 2.

Brain areas	Voxel	T	*P*	MNI coordinates		
Midbrain	91	7.34	< 0.001	19.5	−21	−10.5
Temporal Lobe	796	8.44	< 0.001	−43.5	−43.5	7.5
Middle Occipital Gyrus	26	6.15	< 0.001	−28.5	−88.5	−3
Lingual_R (aal)	23	−6.26	< 0.001	12	−75	1.5
Corpus Callosum	143	7.91	< 0.001	4.5	28.5	1.5
Thalamus_L (aal)	10	6.09	< 0.001	−15	−22.5	3
Sub-lobar	503	9.01	< 0.001	6	−34.5	12
Sub-Gyral	390	7.14	< 0.001	−25.5	−51	13.5
Insula_R (aal)	57	5.99	< 0.001	30	21	15

MNI is the abbreviation of Montreal Neurological Institute.

### Changes of cerebrospinal fluid in different hydration states

**FIGURE 4:**
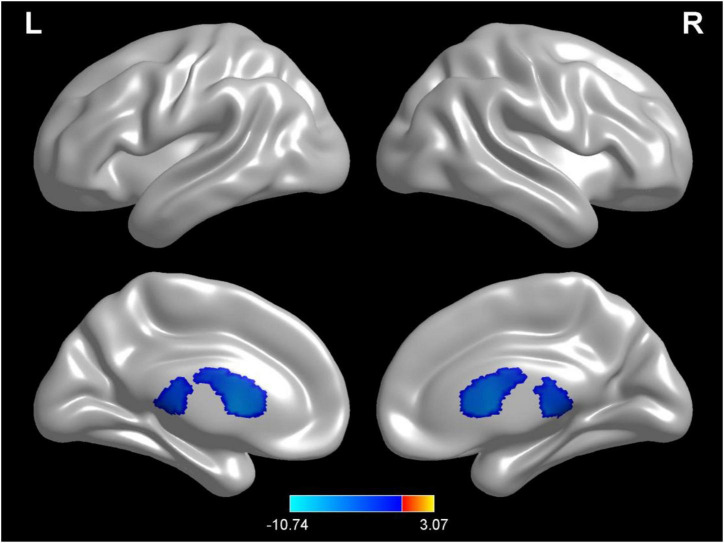
Regional changes on the voxel-based three-dimensional displayed brain cerebrospinal fluid among participants when compared with test 1 in baseline hydration state and test 2 in dehydration state. Warm colors mean that the cerebrospinal fluid density among participants in the baseline hydration test was higher than that in the dehydration test; cold colors mean that the cerebrospinal fluid density among participants in the baseline hydration test was lower than that in the dehydration test; *p* < 0.05 after false-discovery rate correction, voxel threshold of cluster > 10.

**FIGURE 5:**
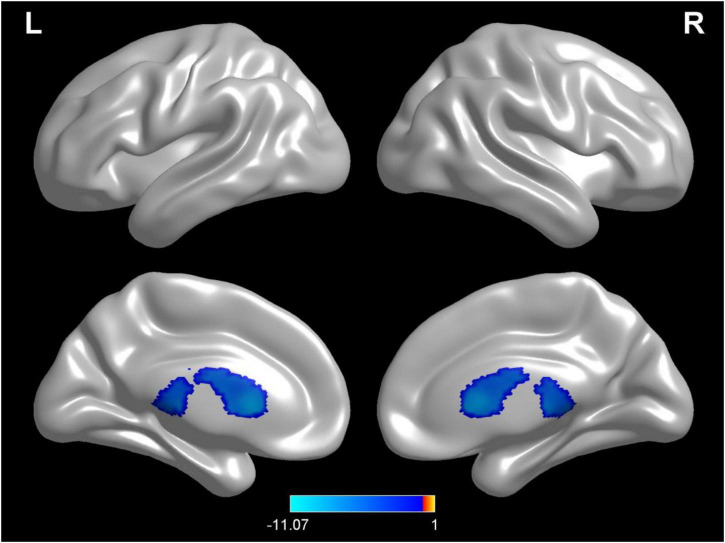
Regional changes on the voxel-based three-dimensional displayed brain cerebrospinal fluid among participants when compared with test 3 in rehydration state and test 2 in dehydration state. Warm colors mean that the cerebrospinal fluid density among participants in the rehydration test was higher than that in the dehydration test; cold colors mean that the cerebrospinal fluid density among participants in the rehydration test was lower than that in the dehydration test; *p* < 0.05 after false-discovery rate correction, voxel threshold of cluster > 10.

Compared with test 1 for the baseline, participants in a dehydration state after 36 hours of water deprivation in test 2 had a higher cerebrospinal fluid density (voxel = 5118; T = −10.74; MNI coordinates: −24, −40.5,13.5; *p* < 0.001) (Figure 4). Compared with test 2, participants in a rehydration state in test 3 had a lower cerebrospinal fluid density (voxel = 5342; T = −11.07; MNI coordinates: 6, 16.5,7.5; *p* < 0.001) (Figure 5).

### Changes in brain homogeneity in different hydration states

**FIGURE 6:**
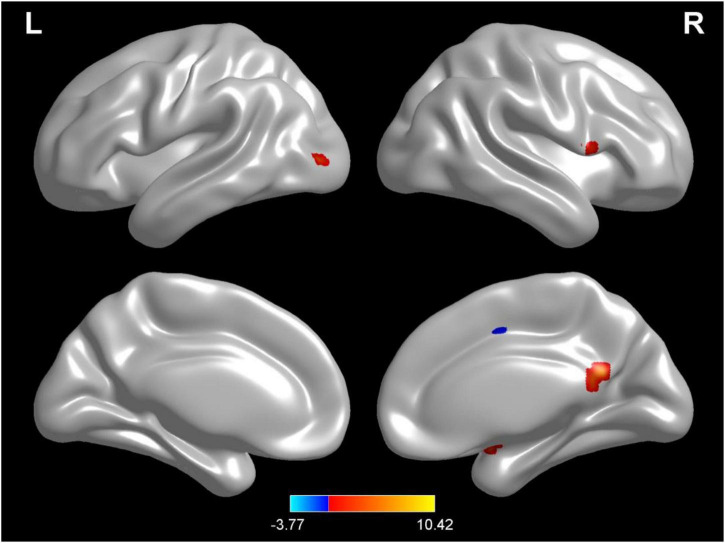
Regional changes on the voxel-based three-dimensional displayed brain regional homogeneity (ReHo) among participants when compared with test 1 in baseline hydration state and test 2 in dehydration state. Warm colors mean that the ReHo value among participants in the baseline test was higher than that in the dehydration test; cold colors mean that the ReHo value among participants in the baseline test was lower than that in the dehydration test; *p* < 0.05 after false-discovery rate correction, voxel threshold of cluster > 10.

Compared with test 1 for baseline, participants in a dehydration state after 36 hours of water deprivation in test 2 had lower ReHo values in the right amygdala, left occiput median, right lingual, opercula part of right inferior frontal gyrus, and right precuneus and a higher ReHo value in the right supplementary activity area (*p* < 0.01) (Table 4 and Figure 6).

**TABLE 4:** Differences in brain regional homogeneity (ReHo) among participants in test 1 for the baseline state with participants in the dehydration state in test 2.

Brain areas	Voxel	T	*P*	MNI coordinates		
Amygdala_R (aal)	8	6.15	< 0.001	24	6	−15
Occipital_Mid_L (aal)	5	4.78	0.001	−39	−84	3
Lingual_R (aal)	10	5.45	< 0.001	6	−57	3
Frontal_Inf_Oper_R (aal)	7	10.42	< 0.001	57	12	6
Precuneus_R (aal)	20	5.74	< 0.001	6	−54	21
Supp_Motor_Area_R (aal)	5	–3.77	0.003	12	3	48

MNI is the abbreviation of Montreal Neurological Institute.

**FIGURE 7:**
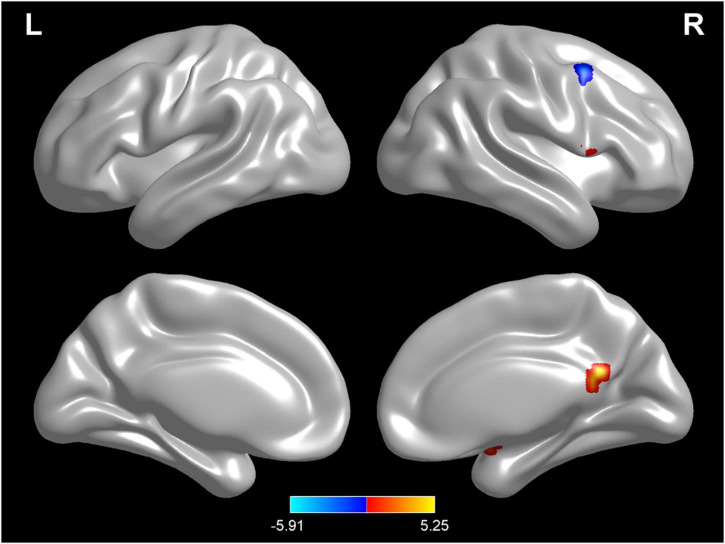
Regional changes on the voxel-based three-dimensional displayed brain regional homogeneity (ReHo) among participants when compared with test 3 in rehydration state and test 2 in dehydration state. Warm colors mean that the ReHo value among participants in the rehydration test was higher than that in the dehydration test; cold colors mean that the ReHo value among participants in the rehydration test was lower than that in the dehydration test; *p* < 0.05 after false-discovery rate correction, voxel threshold of cluster > 10.

Compared with test 2, participants in a rehydration state after water supplementation in test 3 had higher ReHo values in the right amygdala, right lingual, opercula part of right inferior frontal gyrus, and right precuneus and lower ReHo values in the left cerebellopontine area 1 and middle frontal gyrus (*p* < 0.01) (Table 5 and Figure 7).

**TABLE 5:** Differences in brain regional homogeneity (ReHo) among participants in test 3 for the rehydration state with participants in the dehydration state in test 2.

Brain areas	Voxel	T	*P*	MNI coordinates		
Cerebelum_Crus1_L (aal)	7	–5.91	< 0.001	−39	−60	−33
Amygdala_R (aal)	6	2.81	0.016	24	6	−18
Lingual_R (aal)	6	4.00	0.002	9	−54	6
Frontal_Inf_Oper_R (aal)	6	3.64	0.004	57	12	6
Precuneus_R (aal)	20	5.25	0.002	6	−51	21
Middle Frontal Gyrus	6	–3.49	0.005	39	6	51

MNI is the abbreviation of Montreal Neurological Institute.

## Discussion

Currently, most studies focus on the effects of Alzheimer’s syndrome, ischemic brain injury, epileptic encephalopathy, and other clinical diseases on the brain’s structure and the connectivity of brain nodes. However, studies about the effects of hydration on brain structure and the connectivity of brain nodes among healthy adults are scarcely reported. In the study, the effects of hydration on brain regional density and homogeneity were measured using the method of MRI.

It was shown that dehydration increased cerebrospinal fluid density. Rehydration caused an extensive increase in gray matter density and white matter density in some specific brain regions. As one indicator of the connectivity of brain nodes, the ReHo value was also affected by the state of hydration. In China, there have been no other studies about the effects of hydration on the brain’s structure and the connectivity of brain nodes. A few related studies have been conducted in some other countries. The results of one study suggested that dehydration induced by a 16 hour period of fluid restriction reduced the total brain volume, and brain volume was restored following rehydration (16). In a study conducted among sight-active men participants, it was found that dehydration with 2.9% body mass loss induced by intermittent exercise in a warm environment caused reductions in cerebrospinal fluid (27). In two other related studies, the results showed that ventricular volume changes under a hypohydration state in terms of 1.7 to 2.9% body mass loss, but brain volume did not change (28, 29). With ten trained endurance males aged 23 years old as participants, one study found that hypohydration at 3% of body mass loss induced by running on a treadmill reduced total brain volume (30). In one study, MRI scans were also conducted to explore the mechanisms of an acute dehydration state among participants, and it was found that there was an expansion of the ventricular system with the largest change appearance in the left lateral ventricle, which may induce the short-term changes of cognitive performances controlled by the brain. In another study with the method of brain MRI scans, it was suggested that blood-oxygen-level-dependent (BOLD) responses in the fronto-parietal increased and lateral ventricle were enlarged in acute dehydration induced by a thermal exercise protocol (29). However, in a study with twenty healthy volunteers, brain MRIs were scanned in three conditions: a baseline scan, a scan after hydration when consuming 3L of water over 12 h, and a scan after dehydration after overnight fasting for 9 h. Additionally, it was found that brain volume and brain total water content were not substantially affected (17). The ReHo value of regional homogeneity was usually used to evaluate spontaneous neural activity during the resting state and can be used to explore the connectivity of brain nodes and cognitive performances (31). One study demonstrated that changes in ReHo were correlated with changes in cognitive performance in some circumstances (32). The mechanism of the effects of hydration on brain structure and the connectivity of brain nodes may be explained by the following reasons. Dehydration is usually accompanied by hypovolaemia, which may cause an increase in the ventricular system volume and a reduction in brain volume (19, 28, 33, 34). Serum osmolality induced by acute dehydration could produce an osmotic gradient, resulting in an increased diffusion of intracellular water stores into extracellular space. The changes cause shrinkage of cells, particularly astrocytes, which have a vital role in the transport of water, and thus leads to ventricular system expansion (19).

Adverse health effects and related symptoms of mild and moderate dehydration in daily life often do not receive enough attention. In this study, the changes in brain regional density and homogeneity under different hydration states were discovered.

This study has some strengths and weaknesses. Referring to the method of inducing dehydration, dehydration can be induced by heat stress, fluid restriction, exercise, diuretics, or combinations of the above methods in current studies. However, some methods of inducing dehydration may affect brain regional density and homogeneity, such as heat stress and exercise. In this study, water deprivation and supplementation were used to induce changes in hydration states among participants, which may be more meaningful in exploring the effects of hydration on brain structures clearly. In addition, it is also very important to ensure the quality control during water deprivation. The osmolality of urine during the period of water deprivation was continuously monitored to explore the changing trend of hydration state and to verify the adherence of participants, which showed that the study had restricted and high quality control. In some studies, the objective physiological and biochemical indexes are not used to monitor the quality control during water deprivation. In consideration of weakness, gender differences and the effects of long-term water intervention on brain regional density and homogeneity were not studied. In addition, this is a pilot self-control trial to explore the effects of dehydration and rehydration on brain regional density and homogeneity. Randomized controlled design studies could obtain more effective results and reveal scientifically effects of hydration state on brain structure and function more clearly and accurately. In this study, only brain regional density and homogeneity was analyzed. Some other indexed such as brain volume and blood oxygen level dependent were not analyzed, more comprehensive indexes would be helpful to explore the effective of hydration on brain structure and function. Based on this pilot self-control trial, more high-quality research and analysis can be carried out in the future.

## Conclusion

In summary, dehydration state increased cerebrospinal fluid density, decreased brain regional homogeneity. Rehydration state increased brain gray matter and white matter density widespreadly, and in-creased brain regional homogeneity. Maintaining a normal hydration state through sufficient water intake is helpful in maintaining brain regional density and homogeneity.

## Data availability statement

The raw data supporting the conclusions of this article will be made available by the authors, without undue reservation.

## Ethics statement

The study protocol and instruments were reviewed and approved by the Ethical Review Committee of Chinese Nutrition Society on November 10, 2015. The code of identification is CNS-2015-001. The study was conducted in accordance with the guidelines of the Declaration of Helsinki. Prior to the conduction of the study, all participants read and signed informed consent voluntarily. The patients/participants provided their written informed consent to participate in this study.

## Author contributions

NZ, SD, and GM: conceptualization. JZ: data curation. NZ: formal analysis. SD: funding acquisition. NZ and JZ: investigation. NZ, SD, and JZ: methodology and writing—original draft. NZ, JZ, SD, and GM: project administration. SD and GM: supervision. GM: writing—review and editing. All authors were involved in the manuscript revision and have approved this final version, agreed to authorship and order of authorship for this manuscript, and appropriate permissions and rights to the reported data.

## Footnotes

1
http://www.fil.ion.ucl.ac.uk/spm

2
http://people.cas.sc.edu/rorden/mricron/index.html

3
https://helab.bnu.edu.cn/

4
http://rfmri.org/dpabi
