# Supplementary material for: Dehydration and rehydration affect brain regional density and homogeneity among young male adults, determined via magnetic resonance imaging: A pilot self-control trial
Source: Front Nutr. 2022 Sep 23;9:906088. doi: 10.3389/fnut.2022.906088 (PMC9539665; doi:10.3389/fnut.2022.906088)
Supplement: Supplementary file 1 [file Data_Sheet_1.docx]

| Table S1. Characteristics in studies related to the effects of hydration on brain structure and function | | | | | | | | |
| --- | --- | --- | --- | --- | --- | --- | --- | --- |
| Author | Year | Study type | Participants | Sample size | Method of establishing hydration modeling | Method of evaluating brain structure and function | Results | Conclusion |
| Kempton et al[1] | 2011 | Cross-over repeated measures design | Healthy adolescents , mean age 16.8, five females | 10 | Dehydration: thermal exercise protocol | Functional magnetic resonance imaging | Lateral ventricle enlargement correlated with the reduction in body mass(*r*=0.77, *P*=0.01) | Reduced water intake may adversely impact executive function. |
| Wang et al[2] | 2014 | Self-controlled study | Healthy young adults (age 21-32 years) | 10 | Dehydration: fasting >14 hours; euhydration: consuming 1.5L of water | Magnetic resonance imaging | A decrease in CSA following dehydration was detected by both measurement methods, with a mean change of -0.654% (SD=0.778, P<0.05) and -0.650% (SD =1.071, P<0.05) for the first and second methods, respectively. | Dehydration can confound spinal cord cross-sectional area. |
| Duning et al[3] | 2004 | Self-controlled study | Healthy adults (age 22-32 years,9 men and 11 women) | 20 | Dehydration: thirsting for 16 hours; euhydration: drinking 1.5 L of mineral water | Magnetic resonance imaging | Dehydration and rehydration can significantly change brain volume: lack of fluid intake for 16 hours decreased brain volume by 0.55% (SD, 0.69), and after rehydration total cerebral volume increased by 0.72% (SD, 0.21). | Dehydration and rehydration had effects on brain structure. |
| Meyers et al[4] | 2016 | Self-controlled study | 11 male, 9 female, mean age 31, range 21–57 years | 20 | Euhydration: consuming 3L of water over 12 hours；dehydration: overnight fasting of 9 hours | Magnetic resonance imaging | No measurable change was found in  total water content (within any brain region) or brain volume between hydration states | Brain total water content and brain volumes are not substantially affected under different hydration status. |
| Streitbürger et al[5] | 2012 | Self-controlled study | Healthy young adults (3 female; mean age 24.7 years) | 6 | Euhydration: consuming 3-4L water；dehydration: drinking 150 ml of water per day and had to avoid meals with a high fluid content in two days | Magnetic resonance imaging | A significant decrease of GM and WM volume associated with dehydration was found in various brain regions, most prominently, in temporal and sub-gyral parietal areas, in the left inferior orbito-frontal region, and in the extra-nuclear region. | Hydration had effects on gray matter (GM), white matter (WM) and ventricular volume. |
| Wittbrodt et al[6] | 2016 | Self-controlled study | Nine physically active adults (four male, five female; 23.9 ± 9.3 years) | 9 | Euhydration: 2.5 h intermittent walking in the heat with water ingestion to match sweat loss；Hypohydration: 2.5 h intermittent walking in 45ºC, 15% RH | Magnetic resonance imaging | Hypohydration tended to decrease (p = 0.06) subcortical grey matter by -1.4% (ES: 0.76) and increase (p < 0.05) ventricular volume by 12.5% (ES: 1.6) and cerebrospinal fluid volume by 13.7% (ES: 1.7) compared to euhydration. Euhydration had lower ventricular and cerebrospinal fluid volumes (-5.1%, ES: 0.72; -6.2%, ES: 1.13) but higher intracranial volume (1.4%; ES: 0.84). | Hypohydration of ~3% body mass loss decreased intracranial volume and may reduce subcortical grey matter volume; expanded ventricle and cerebrospinal fluid volumes. |
| Watson et al[7] | 2010 | Self-controlled study | Physically active men (Mean ± SD age 26 ± 4 years) | 8 | Euhydration: ingestion of 500 ml of plain water；Hypohydration: a series of 10 min periods of cycle exercise at an intensity corresponding to 60 % of VO_2_ peak | Magnetic resonance imaging | Brain volume was not influenced by hypohydration (0.2 ± 0.4 %; ES 0.2; P = 0.310). | Brain volume remains unchanged in response to moderate hypohydration |
| Dickson et al[8] | 2005 | Self-controlled study | Healthy subjects | 6 | Euhydration: ingestion of 500 ml of plain water；Hypohydration: a series of 10 min periods of cycle exercise at an intensity corresponding to 60 % of VO_2_ peak | Magnetic resonance imaging | A correlation between the degree of dehydration and the change in ventricular volume (r = 0.932, p = 0.007) was found. | The changes in ventricular volume caused by dehydration were much larger than those seen in day-to-day fluctuations in a normally hydrated healthy control subject. |
| Kempton et al[9] | 2009 | Self-controlled study | Male volunteers,mean age 23.8 years | 7 | Dehydration: 90 min thermal exercise protocol | Magnetic resonance imaging | There was a significant correlation between loss in body mass and third ventricular volume increase (r=0.79, P=0.03) | Ventricular expansion occurs following acute dehydration |
| Tan et al[10] | 2017 | Randomized, counterbalanced trials | Endurance-trained sportsmen (mean±sd: age 23.3±1.1 years) | 10 | Hypohydration: -3% body mass by running on a treadmill at 65% VO_2_ peak in a 25°C environment | Magnetic resonance imaging | Fold changes in total brain volume were lower in HH trials than EU trials (EU: 1.007, HH: 0.993; p=0.003). | Total brain volume is reduced with hypohydration but notably the functional activity of the brain is well-preserved in these endurance athletes. |

References

[1] Matthew J Kempton, Ulrich Ettinger, Russell Foster, Steven C R Williams, Gemma A Calvert, Adam Hampshire, Fernando O Zelaya, Ruth L O'Gorman, Terry McMorris, Adrian M Owen, and M.S. Smith, Dehydration affects brain structure and function in healthy adolescents. Hum Brain Mapp 32 (2011) 71-79.

[2] C Wang, R C Tam, E Mackie, D K B Li, and A.L. Traboulsee, Dehydration affects spinal cord cross-sectional area measurement on MRI in healthy subjects. Spinal Cord 52 (2014) 616-620.

[3] T Duning, S Kloska, O Steinsträter, H Kugel, W Heindel, and S. Knecht, Dehydration confounds the assessment of brain atrophy. Neurology 64 (2005) 548-50.

[4] Sandra M Meyers, Roger Tam, Jimmy S Lee, Shannon H Kolind, Irene M Vavasour, Emilie Mackie, Yinshan Zhao, Cornelia Laule, Burkhard Mädler, David K B Li, Alex L MacKay, and A.L. Traboulsee, Does hydration status affect MRI measures of brain volume or water content? J Magn Reson Imaging 44 (2016) 296-304.

[5] Daniel-Paolo Streitbürger, Harald E Möller, Marc Tittgemeyer, Margret Hund-Georgiadis, Matthias L Schroeter, and K. Mueller, Investigating structural brain changes of dehydration using voxel-based morphometry. Plos One 7 (2012) e44195.

[6] M. Wittbrodt, J.C. Mizelle, L.A. Wheaton, M.N. Sawka, and M.L. Millard-Stafford, Impact of Hypohydration and Exercise-Heat Stress on Brain Structure in Men and Women, American College of Sports Medicine Meeting, 2016.

[7] Phillip Watson, Kay Head, Alain Pitiot, Peter Morris, and R.J. Maughan, Effect of exercise and heat-induced hypohydration on brain volume. Med Sci Sports Exerc 42 (2010) 2197.

[8] J M Dickson, H M Weavers, N Mitchell, E M Winter, I D Wilkinson, E J R Van Beek, J M Wild, and P.D. Griffiths, The effects of dehydration on brain volume - preliminary results. Int J Sports Med 26 (2005) 481-485.

[9] Matthew J Kempton, Ulrich Ettinger, Anne Schmechtig, Edward M Winter, Luke Smith, Terry McMorris, Iain D Wilkinson, Steven C R Williams, and M.S. Smith, Effects of acute dehydration on brain morphology in healthy humans. Hum Brain Mapp 30 (2019) 291-298.

[10] Xiang Ren Tan, S.C. Mary, T. Kok, and T.W. Soong, Effects Of Exercise-induced Hypohydration On Brain Structure And Function, A MRI Study. Med Sci Sports Exerc 49 (2017) 824.

| Table S2. Anthropometric measurements of subjects | | | |
| --- | --- | --- | --- |
|  | Baseline test | Dehydration test | Rehydration test |
| Height (cm) | 176.0±5.5 | 176.0±5.5 | 176.0±5.5 |
| Weight (kg) | 68.0±10.9 | 67.2±10.5 | 68.4±10.3 |
| BMI (kg/m^2^) | 21.9±3.0 | 21.6±2.9 | 22.0±2.8 |
| Blood glucose (mmol/L) | 4.3±0.3 | 4.5±0.4 | 4.9±0.2 |
| Blood pressure |  |  |  |
| Systolic pressure (mmHg) | 114.3±7.3 | 111.6±7.9 | 115.9±8.4 |
| Diastolic pressure (mmHg) | 75.1±7.8 | 73.9±6.5 | 75.4±5.0 |

| Table S3. Total fluid from food, 24-h urine volume, void number of subjects on day 3 | | | |
| --- | --- | --- | --- |
| Subjects ID | Total fluid from food (ml) | 24-h urine volume (ml) | Void number |
| 1 | 1029 | 999 | 7 |
| 2 | 1051 | 746 | 6 |
| 3 | 912 | 832 | 3 |
| 4 | 959 | 745 | 3 |
| 5 | 1043 | 722 | 6 |
| 6 | 1130 | 1031 | 6 |
| 7 | 611 | 552 | 3 |
| 8 | 962 | 941 | 4 |
| 9 | 752 | 668 | 3 |
| 10 | 1044 | 883 | 5 |
| 11 | 936 | 662 | 2 |
| 12 | 839 | 811 | 7 |
| Average±Standard Deviation | 939±146 | 799±145 | 5±2 |


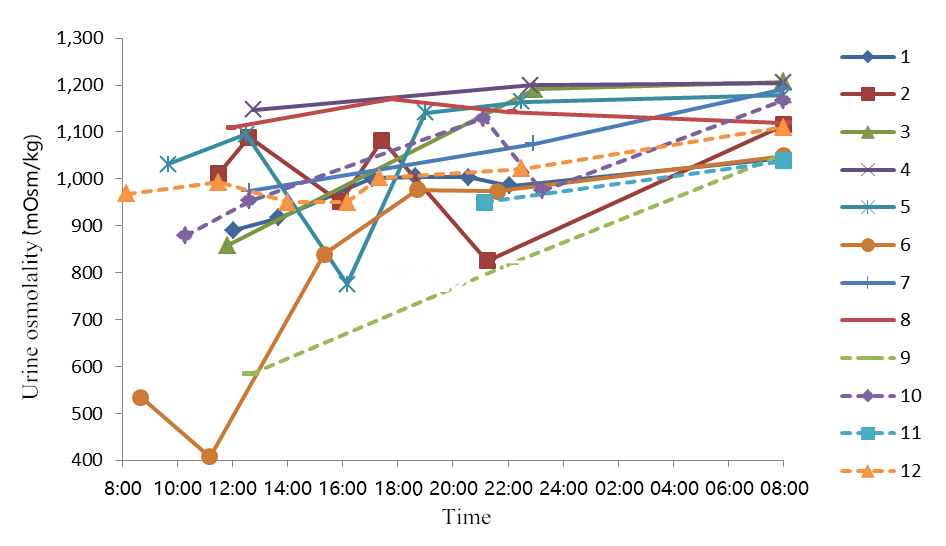


Figure S1. Changing trend of urine osmolality for each subjects on day 3
